# Systematic review of exercise therapy in the management of post-thrombotic syndrome

**DOI:** 10.1177/02683555221129738

**Published:** 2022-09-26

**Authors:** Sara Jasionowska, Benedict R H Turner, Matthew Machin, Sarah Onida, Adam M Gwozdz, Joseph Shalhoub, Alun H Davies

**Affiliations:** 1 Section of Vascular Surgery, Department of Surgery and Cancer, 170714 Imperial College London, London, UK; 2 Imperial Vascular Unit, 8946 Imperial College Healthcare NHS Trust, London, UK

**Keywords:** Post-thrombotic syndrome, exercise, exercise therapy, deep vein thrombosis, chronic venous disease

## Abstract

**Objectives:**

Exercise improves haemodynamic parameters in patients with chronic venous disease. There is a paucity of evidence on its effect in post-thrombotic syndrome (PTS). The aim of this systematic review is to assess the impact of exercise in PTS.

**Methods:**

Adhering to PRISMA guidelines and following PROSPERO registration (CRD42021220924), MEDLINE, Cochrane Library, EMBASE database, and trial registries were searched on 19th May 2022.

**Results:**

One article met the inclusion criteria and a narrative synthesis was carried out. The included randomised controlled trial reported a between-group mean difference of 4.6 points (*p* = .027) in the VEINES-QOL score and −2.0 points (*p* = .14) in the Villalta score, in favour of exercise therapy. The statistical significance threshold was not reached.

**Conclusion:**

Data on exercise in PTS remains sparse but exercise appears to be a safe intervention. In the context of this literature, a potential future trial and outcome reporting measures are suggested.

## Introduction

Post-thrombotic syndrome (PTS) is a chronic consequence of deep vein thrombosis (DVT), characterised by symptoms of chronic venous insufficiency including leg pain, swelling, heaviness and, if severe, venous ulceration.^1^ The annual incidence of DVT in the UK is 1–2 per 1000 people and an estimated 52.6% of patients develop PTS within a few months to a few years after DVT. It is estimated that 5–10% of patients develops severe PTS with venous ulceration.^2^

PTS is a burdensome condition resulting in significantly worse quality of life and disability compared to chronic lung disease, angina and osteoarthritis.^3^ It is also associated with a considerable healthcare burden with an annual direct cost of at least $200 million in the USA, mostly attributed to the intensive wound care and other medical care required.^4^

The condition results from damage to venous valves causing valvular reflux. Additionally, incomplete thrombus clearance and venous scarring result in impaired venous return. This subsequently results in venous hypertension with the clinical signs and symptoms of PTS such as redness, swelling, heaviness and pain.^5,6^

Currently, there are limited evidence-based treatment options for PTS; management is centred on compression therapy.^2^ Therefore, it is crucial to identify new adjunctive treatment modalities that may reduce patient morbidity and improve quality of life.

Exercise therapy has been shown to be effective in treating arterial claudication and has the potential to improve outcomes in PTS.^7^ Potential mechanisms through which exercise may alleviate the symptoms and severity of PTS include improved endurance due to better aerobic capacity, reduced swelling and discomfort from improved calf muscle pump function, and reduced muscular effort due to increased muscular strength.^7^

However, there is a paucity of evidence on the use of exercise in the management of PTS and, thus far, no recommendations have been proposed by the UK National Institute for Health and Care Excellence.

The primary aim of this systematic review is to assess the benefit of exercise in PTS, focusing on improvement of quality of life and severity of signs and symptoms. Secondary aims are to assess improvement in leg strength, flexibility, endurance, compliance with therapy and adverse events.

## Methods

This systematic review was performed with adherence to Preferred Reporting Items for Systematic Reviews and Meta-Analyses (PRISMA) guidelines.^8^ A pre-defined study protocol was registered on PROSPERO (CRD42021220924).

### Search strategy

The literature search was carried out on 19th May 2022. The search strategy consisted of 37 terms for exercise interventions and PTS (Supplementary Table 1). Databases reviewed included MEDLINE, EMBASE and The Cochrane Library. Trial registries including ClinicalTrial.gov, European Union Clinical Trials and International Standard Randomised Controlled Trial Number Registry were searched for grey literature. The searches were performed on an ‘All Fields’ basis. There were no restrictions regarding date of publication, language or study design. After the primary search, the references of included articles and relevant systematic reviews were reviewed for any other eligible articles.

### Eligibility criteria

Studies eligible for inclusion had the following characteristics:

1. The use of exercise in the management of PTS.
2. Articles included details of exercise therapy (e.g. supervised, non-supervised, physiotherapy, etc.)
3. Outcome of severity of PTS as measured by Villalta scale.
4. A follow-up period to assess the outcomes of exercise intervention.
5. Randomised and non-randomised studies reporting on cohorts of ≥10 participants.

Excluded articles had the following characteristics:
1. Chronic venous disease not caused by previous venous thrombosis.
2. Non-human studies, case reports, full-text not available in English, conference abstracts, non-original research, including narrative review articles, duplicate publications.

### Article screening

The articles identified during the searches were screened against the inclusion criteria by two reviewers (SJ, BT) independently using EndNote X9 and Covidence. Initial screening was based on title and abstract. Following this, full-text review was performed for all eligible articles by two authors (SJ, BT). Any discrepancies were mediated by an independent third reviewer (MM).

### Data extraction

Two reviewers (SJ, BT) extracted the data using a pre-defined template in Microsoft Excel 2013. Data retrieved included first author, title, year of publication, location, number of participants, intervention arm, control arm standard, exercise programme used and its duration, length of follow-up, definition of PTS used, change in PTS severity, change in leg muscle strength and flexibility and adverse events (if reported). The third reviewer (MM) mediated any discrepancies in the data extraction.

### Quality assessment

Grading of Recommendations Assessment, Development and Evaluation (GRADE) assessment was undertaken on the online GradePro platform to assess the quality of the extracted data.

### Data synthesis

The RevMan 5 platform was planned to be used for data pooling, however, the data extracted were insufficient for quantitative synthesis. Thus, a narrative synthesis was performed.

## Results

**Figure 1.**
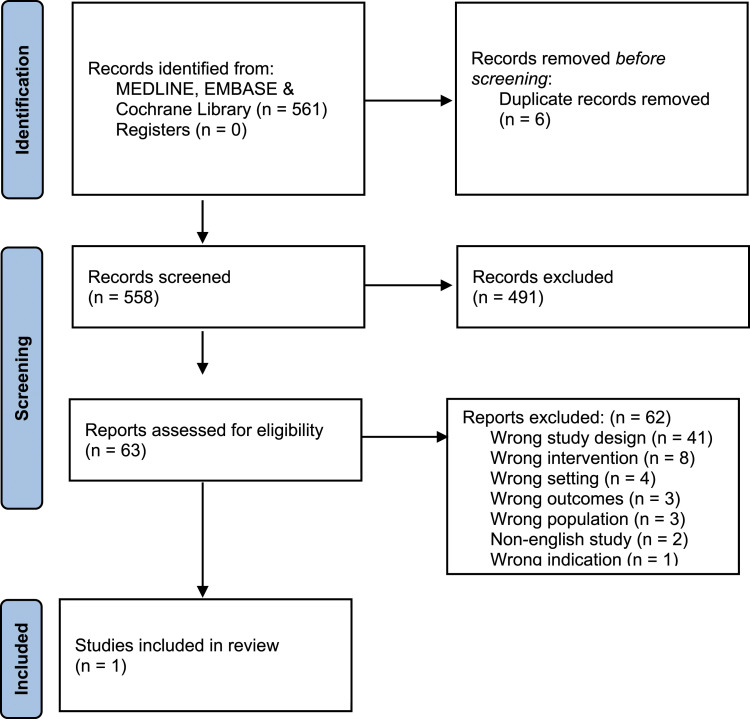
PRISMA flow diagram demonstrating article screening and subsequent inclusion.

Searches of online databases and trial registries returned 561 articles, after full-text exclusions were completed, a narrative synthesis was performed on a single eligible article. Details of the screening process are presented in Figure 1.

### Study characteristics

The only study that met the inclusion criteria was a prospective, randomised controlled trial published in 2011 comparing an exercise therapy group to a control group receiving a single educational session.^7^ The study was a two-centre Canadian pilot trial assessing the feasibility and effectiveness of a multicentre-based 6 months’ exercise therapy programme to treat PTS. Forty-three patients met the inclusion criteria but four dropped out of the exercise group, resulting in 39 patients completing the 6-month follow-up. Two patients dropped out because of time constraints and two for unknown reasons. All patients included in the study had PTS defined as Villalta score of 5 or more and an ultrasound diagnosed DVT at least 6 months previously. Patients who had contraindications to exercise therapy (including lower extremity arthritis, angina, or severe lung disease), pregnant patients and patients with open venous leg ulcers were excluded from the study. Additionally, all patients who failed exercise stress test for reasons other than fatigue were excluded.

At baseline, the exercise group had a higher proportion of males (52.4% vs 36.4%) and participants who reported high levels of regular physical activity (45% vs 22.7%). The mean age was also lower in the exercise group (44.9 years old vs 48.14 years old). Even though the mean Villalta score was similar in both groups (10.05 in exercise group vs 9.36 in control group), subjects in the exercise group were more likely to have severe PTS (19% vs 4.5%), despite randomisation. The mean time since the most recent DVT was 54.1 months in the exercise group and 38.8 months in the control group. However, the statistical significance of the differences in the baseline characteristics was not reported.

As the benefit of compression hosiery in PTS is unclear, the authors documented stocking use but did not request that patients wear compression during the trial.^9^ At baseline, 57.1% of patients allocated to the exercise group and 72.7% in the control group reported wearing compression stockings. At 6 months, the reported use of compression stockings was 52.4% and 68.2% of subjects in the exercise and control groups, respectively. The grade and type of compression stockings were not documented. Further study characteristics are summarised in Table 1.

**Table 1.** Study characteristics for included article.

Study	Number of participants	Design	Study traits	Exercise intervention	Compliance	Outcome assessment	Follow-up
Kahn et al. 2011, Canada	39; 17 exercise vs 22 control	Assessor-blinded prospective RCT	Age 44.9 years vs 48.14 years, severe Villalta score 19% vs 4.5%, time from the last DVT 54.1 vs 38.8 months in exercise vs control, respectively	6-months exercise programme including supervised and non-supervised aerobic, stretching and strength components	61.9% in exercise attended >60% of training sessions	Villalta score, VEINES-QOL, heel lifts, leg muscles flexibility, time on treadmill	6 months

### Quality of evidence

The GRADE assessment was performed for the improvement in quality for life (VEINES-QoL score) and Villalta score change. In light of the inconsistency, imprecision, and indirectness of the results, the outcomes were graded as very low certainty.

### Risk of bias assessment

The article was evaluated using the Cochrane Risk of Bias tool. The risk of selection bias from random sequence generation was low as a computerised sequence were used.

Group allocation was performed by a non-blinded assessor after the baseline assessment was completed, resulting in a low risk of bias from allocation concealment. The study was at high risk of performance bias as neither the participants nor researchers who administered the treatment were blinded and there were systematic differences in the care received by each group. Outcome assessors were blinded and thus, detection bias was low. Modified intention-to-treat analysis that included all participants with data at baseline and 6 months was performed. Attrition was unbalanced between the two groups with no participants lost to follow-up in the control group and four lost to follow-up in the exercise group and hence, the risk of attrition bias was high. The study adhered to the pre-registered protocol and the risk of bias from selective reporting was low. Full details are presented in Supplementary Table 2.

### Exercise intervention

The article compared a structured 6-month exercise therapy to a single educational session. The exercise programme included physiotherapist supervised and unsupervised strengthening, stretching and aerobic components. Patients were asked to attend a total of 15 one-to-one sessions with a trainer, with duration and frequency decreasing incrementally on a weekly basis. The non-supervised component involved the strengthening programme 3–4 times per week, stretching 7 times a week and aerobic programme for 60–120 min per week. The control intervention was a one-hour educational slide presentation on PTS followed by phone calls at one, two, four and five months to assess general well-being and leg symptoms.

### Quality of life

Change in Venous disease-specific quality of life score at 6 months was assessed by the validated VEINES-QOL questionnaire, with a difference of three points considered clinically relevant.^10^ The mean difference in score from baseline to 6 months was 6.0 (SD 5.1) for the exercise group and 1.4 (SD 7.2) for the control group, with a between-group difference of 4.6 points (95% CI 0.54–8.7, *p* = .027) in favour of exercise therapy.

### Severity of PTS signs and symptoms

The mean difference in Villalta score from baseline to 6 months was −3.6 (SD 3.7) in the exercise group and −1.6 (SD 4.3) in the control patients. The between-group difference was −2.0 points (95% CI –4.6 to 0.6, *p* = .14), showing a greater improvement in symptoms in the exercise group.

### Leg strength

The heel-lift test was used to measure the strength of calf muscles. The Habedometer device was used. The mean difference in maximum number of heel lifts performed from baseline to 6 months was 5.2 lifts in the exercise group and −2.5 lifts in the control group, with a between-group difference of 7.7 lifts (95% CI 0.7–14.7, *p* = .03) in favour of exercise therapy.

### Leg flexibility

Muscle flexibility was measured with a handheld inclinometer. The mean quadriceps flexibility improved by 10.2° in the exercise group and 0.3° in the control group, with a between-group difference of 9.9° (95% CI –1.0–20.7, *p* = .04) favouring exercise therapy. The improvement in the flexibility of hamstring, soleus and gastrocnemius muscles was higher in the exercise group, though the increase in flexibility was much smaller compared to quadriceps flexibility.

### Endurance

Time on treadmill was used as a proxy for exercise capacity. The mean change in treadmill time at 6 months was −0.5 min in exercise group and 0.03 min for control group, with a between-group difference of −0.53 (95% CI −1.76–0.69, *p* = .33) marginally in favour of the control group.

### Compliance

Sixty-two percent of patients reached the pre-specified minimal adherence level set as attendance at >60% of supervised sessions. Compliance with the non-supervised exercise therapy was assessed using a self-reported exercise log. Over 60 percent of participants completed their exercise log during the first five months, and 40% completed it during the last month of the study. Of those who completed their exercise log, the mean number of sessions was 3.1 per week of strengthening training, 5.1 per week of stretching exercise, and 3.8 per week of aerobic exercise with a mean duration of 150.5 min per week.

### Adverse events

No adverse events were reported for participants in either group.

## Discussion

Patients affected by PTS suffer from lifelong debilitating symptoms that considerably reduce their quality of life and can result in serious complications such as venous ulceration in 29% of patients, according to population cohort studies.^3^ Clinical practice guidelines on the management of PTS remain largely inconsistent and fail to cover a wide range of therapeutic options.^11^ Treatment options are sparse and have only been evaluated in low-quality trials. Despite the severe impact of PTS on patients and the lack of evidence-based treatments, there was only one RCT assessing the use of exercise in the management of PTS identified by this review. Even though this systematic review yielded a single paper, it is still a valid review which summarises the most up to date evidence on the effect of exercise in management of PTS which will serve as a foundation for future studies.

Exercise therapy has been shown to be an effective treatment for arterial claudication and may also improve symptoms of PTS. Potential mechanisms include reduced swelling, improved calf muscle strength and increased ankle and knee flexibility.^12^ Additionally, supervised exercise therapy in patients with PTS in the acute setting showed that exercise did not exacerbate symptoms and it may have a role in reducing calf stiffness.^13^

The trial included in this review provides useful data on the feasibility and clinical outcomes of a 6-month exercise programme in a PTS patient cohort. The improvement in symptoms severity and quality of life observed in the exercise group was attributed to increased leg strength and flexibility combined with possible psychobiological mechanisms.

Additionally, compliance with the supervised exercise component was expressed as a percentage of participants who exceeded the pre-specified compliance threshold rather than an absolute value, which makes the estimation of the true treatment effect of exercise as treatment for PTS very challenging. Thus, in future trials the use of absolute values when reporting compliance should be used. The compliance with exercise programmes decreased during the 6th month of the study included in this review and thus, researchers postulated that future studies could investigate the effectiveness of shorter programmes, for example, 3 months.

Future clinical trials should be designed as assessor-blind, randomised trials of at least 3 months duration and a follow-up evaluation at 6 months. A pilot study should be carried out to allow for sample size calculation and adequate powering of the study. The participants should be randomly allocated (1:1) into treatment and control groups. The intervention should consist of physiotherapist supervised and unsupervised components carried out for at least 30 min at least 3 times a week, as reported by previous trials investigating the role of exercise in peripheral arterial disease.^14^ Possible exercise types which should be incorporated into the exercise regime include aerobic training, strengthening and stretching.

The Villalta scale has been reported to be a reliable, valid, and acceptable measure for PTS but a point of criticism has been that it was not developed via formal psychometric analysis and it tends to overcapture mild disease which is not clinically significant.^15^ In future trials, the Villalta scale should be used as a primary outcome measure, in conjunction with a patient-reported venous disease-specific quality of life score.^16^ The secondary outcomes should include changes in exercise capacity, ankle flexibility, lower limb muscle strength and adverse outcomes.

Compliance with the unsupervised component of the exercise therapy should be monitored with validated exercise diaries and reported as an absolute number rather than a proportion of individuals who met the pre-specified compliance threshold.

### Limitations

Only one RCT was included in this review, which highlights that there is a lack of evidence supporting the use of exercise in the management of PTS. Therefore, conclusions that can be drawn on the effectiveness of exercise therapies in treatment of PTS are very limited. Additionally, the included study reported outcomes for a small cohort and the majority of outcomes failed to reach statistical significance.

Furthermore, the cohort of patients investigated in the included study was younger than the average population affected by PTS, which makes the generalisability of data to a wider population difficult.

## Conclusion

This systematic review has highlighted the paucity of evidence on the use of exercise in the management of PTS. Therefore, the effectiveness of exercise therapies in PTS remains unclear.

This review provides an update of the available evidence and offers a basis for future RCTs. Adequately powered single-blinded RCTs are required to provide Level I evidence to support the use of exercise in PTS patients. Supervised exercise programmes may have the potential to offer the support and motivation patients need when exercising and home-based unsupervised programmes may allow patients to exercise at a time convenient for them. Future studies should explore both types of exercise and investigate which options are favoured by patients and result in the highest compliance rates.

## Supplemental Material

Supplemental Material - Systematic review of exercise therapy in the management of post-thrombotic syndrome Click here for additional data file. Supplemental Material for Systematic review of exercise therapy in the management of post-thrombotic syndrome by Sara Jasionowska, Benedict R H Turner, Matthew Machin, Sarah Onida, Adam M Gwozdz, Joseph Shalhoub and Alun H Davies in Phlebology

## References

[1] Eklof B Perrin M Delis K, et al. Updated terminology of chronic venous disorders: the VEIN-TERM transatlantic interdisciplinary consensus document. J Vasc Surg 2009; 49(2): 498–501. 19216970 10.1016/j.jvs.2008.09.014

[2] Kahn S Comerota A Cushman M, et al. The postthrombotic syndrome: evidence-based prevention, diagnosis, and treatment strategies. Circulation 2014; 130(18): 1636–1661. 25246013 10.1161/CIR.0000000000000130

[3] Kahn SR M’Lan CE Lamping DL, et al. The influence of venous thromboembolism on quality of life and severity of chronic venous disease. J Thromb Haemost 2004; 2: 2146–2151. 15613019 10.1111/j.1538-7836.2004.00957.x

[4] Heit J Rooke T Silverstein M, et al. Trends in the incidence of venous stasis syndrome and venous ulcer: a 25-year population-based study. J Vasc Surg 2001; 33(5): 1022–1027. 11331844 10.1067/mva.2001.113308

[5] Haenen J Janssen M van Langen H, et al. The postthrombotic syndrome in relation to venous hemodynamics, as measured by means of duplex scanning and strain-gauge plethysmography. J Vasc Surg 1999; 29(6): 1071–1076. 10359941 10.1016/s0741-5214(99)70248-x

[6] Prandoni P Frulla M Sartor D, et al. Vein abnormalities and the post-thrombotic syndrome. J Thromb Haemost 2005; 3(2): 401–402. 15670059 10.1111/j.1538-7836.2004.01106.x

[7] Kahn S Shrier I Shapiro S, et al. Six-month exercise training program to treat post-thrombotic syndrome: a randomized controlled two-centre trial. Can Med Assoc J 2010; 183(1): 37–44. 21098066 10.1503/cmaj.100248 PMC3017252

[8] Moher D Liberati A Tetzlaff J, et al. Preferred reporting items for systematic reviews and meta-analyses: the PRISMA statement. PLoS Med 2009; 6(7): e1000097. 19621072 10.1371/journal.pmed.1000097 PMC2707599

[9] Kahn S Shapiro S Wells P. Compression stockings to prevent post-thrombotic ayndrome: a randomised placebo-controlled trial. J Vasc Surg 2014; 59(5): 1470.

[10] Lamping D Schroter S Kurz X, et al. Evaluation of outcomes in chronic venous disorders of the leg: development of a scientifically rigorous, patient-reported measure of symptoms and quality of life. J Vasc Surg 2003; 37(2): 410–419. 12563215 10.1067/mva.2003.152

[11] Machin M Salim S Tan M, et al. Surgical and non-surgical approaches in the management of lower limb post-thrombotic syndrome. Expert Rev Cardiovasc Ther 2021; 19(3): 191–200. 33455484 10.1080/14779072.2021.1876563

[12] Padberg F Johnston M Sisto S. Structured exercise improves calf muscle pump function in chronic venous insufficiency: a randomized trial. J Vasc Surg 2004; 39(1): 79–87. 14718821 10.1016/j.jvs.2003.09.036

[13] Kahn S Azoulay L Hirsch A, et al. Acute effects of exercise in patients with previous deep venous thrombosis. Chest 2003; 123(2): 399–405. 12576357 10.1378/chest.123.2.399

[14] McDermott M Ades P Guralnik J. Treadmill exercise and resistance training in patients with peripheral arterial disease with and without intermittent claudication: a randomized controlled trial. J Vasc Surg 2009; 50(1): 234–235. 10.1001/jama.2008.962 PMC3268032 19141764

[15] Lee A Gu C Vedantham S, et al. Performance of two clinical scales to assess quality of life in patients with post-thrombotic syndrome. J Vasc Surg Venous Lymphatic Disord 2021; 9(5): 1257–1265. e2. 10.1016/j.jvsv.2021.01.017 PMC8333181 33548557

[16] Enden T Wik H Kvam A, et al. Health-related quality of life after catheter-directed thrombolysis for deep vein thrombosis: secondary outcomes of the randomised, non-blinded, parallel-group CaVenT study. BMJ Open 2013; 3(8): e002984. 10.1136/bmjopen-2013-002984 PMC3758969 23988361

