# Supplementary material for: Systematic review of exercise therapy in the management of post-thrombotic syndrome
Source: Phlebology. 2022 Sep 26;37(10):695–700. doi: 10.1177/02683555221129738 (PMC9720701; doi:10.1177/02683555221129738)
Supplement: Supplemental Material - Systematic review of exercise therapy in the management of post-thrombotic syndrome [file sj-pdf-1-phl-10.1177_02683555221129738.pdf]

Supplementary table 1. Search strategy carried out on to search online databases.

Search #	Search Terms (MEDLINE and Cochrane)
1	Exercis* OR aerobic OR physical OR strength OR interval OR resistance OR isometric OR strength OR cardiovasc* OR aqua* OR Structured OR Supervi?e*) adj3 (exercis* OR activit* OR therap* OR program* OR train* OR rehab*):mp OR Physiotherapy.mp OR (Sport* or danc* or yoga or pilates or swim*).mp<br><br>MESH: Exercise/ OR Sports/ OR Dancing/ OR Yoga/ OR Rehabilitation
AND	
2	Post-thrombotic syndrome OR postthrombotic disease OR post thrombotic syndrome OR postphlebitic syndrome OR venous stress disorder OR PTS<br>MESH: Postthrombotic syndrome/ OR Postphlebitic syndrome/

	Author's judgement	Support for judgement
Random sequence generation (selection bias)	Low risk	Patients were randomized to exercise training or control via a web-based program (Dacima Software Inc.) that ensured concealment of treatment allocation. Randomization was stratified by study centre. Each study site had two research assistants, one unblinded and one blinded to treatment allocation. The unblinded assistant notified patients of their allocated treatment only after the baseline assessment was completed, interacted with the exercise training facility to arrange appointments for exercise-training patients and administered the control treatment to control patients. The blinded assistant performed the baseline, three-month and six-month assessments of all patients, who were instructed not to reveal their allocated treatment.
Allocation concealment (selection bias)	Low risk	Patients were randomized to exercise training or control via a web-based program (Dacima Software Inc.) that ensured concealment of treatment allocation. Randomization was stratified by study centre. Each study site had two research assistants, one unblinded and one blinded to treatment allocation. The unblinded assistant notified patients of their allocated treatment only after the baseline assessment was completed, interacted with the exercise training facility to arrange appointments for exercise-training patients and administered the control treatment to control patients. The blinded assistant performed the baseline, three-month and six-month

		assessments of all patients, who were instructed not to reveal their allocated treatment.
Blinding of participants and researchers (performance bias)	High risk	Patient were not blinded and neither was the researcher who was administering intervention/control treatment
Blinding of outcome assessment (detection bias)	Low risk	Outcome assessment performed by a blinded researcher
Incomplete outcome data (attrition bias)	High risk	no intention to treat analysis performed, patients who were lost to follow up were not included in the analysis
Selective reporting (reporting bias)	Low risk	Adherence to the original study protocol
Other bias	Low risk	
